# Preventive and curative effect of melatonin on mammary carcinogenesis induced by dimethylbenz[a]anthracene in the female Sprague–Dawley rat

**DOI:** 10.1186/bcr1031

**Published:** 2005-04-29

**Authors:** Véronique Lenoir, Marianne Beau Yon de Jonage-Canonico, Marie-Hélène Perrin, Antoine Martin, Robert Scholler, Bernard Kerdelhué

**Affiliations:** 1 Laboratoire de neuroendocrinologie, CNRS-FRE2718, UFR Biomédicale des Saints-Pères, Université René Descartes, Paris; 2 Service Central d'Anatomie et de Cytologie Pathologique, Hôpital Avicenne, Bobigny, France

## Abstract

**Introduction:**

It has been well documented that the pineal hormone, melatonin, which plays a major role in the control of reproduction in mammals, also plays a role in the incidence and growth of breast and mammary cancer. The curative effect of melatonin on the growth of dimethylbenz [a]anthracene-induced (DMBA-induced) mammary adenocarcinoma (ADK) has been previously well documented in the female Sprague–Dawley rat. However, the preventive effect of melatonin in limiting the frequency of cancer initiation has not been well documented.

**Methods:**

The aim of this study was to compare the potency of melatonin to limit the frequency of mammary cancer initiation with its potency to inhibit tumor progression once initiation, at 55 days of age, was achieved. The present study compared the effect of preventive treatment with melatonin (10 mg/kg daily) administered for only 15 days before the administration of DMBA with the effect of long-term (6-month) curative treatment with the same dose of melatonin starting the day after DMBA administration. The rats were followed up for a year after the administration of the DMBA.

**Results:**

The results clearly showed almost identical preventive and curative effects of melatonin on the growth of DMBA-induced mammary ADK. Many hypotheses have been proposed to explain the inhibitory effects of melatonin. However, the mechanisms responsible for its strong preventive effect are still a matter of debate. At least, it can be envisaged that the artificial amplification of the intensity of the circadian rhythm of melatonin could markedly reduce the DNA damage provoked by DMBA and therefore the frequency of cancer initiation.

**Conclusion:**

In view of the present results, obtained in the female Sprague–Dawley rat, it can be envisaged that the long-term inhibition of mammary ADK promotion by a brief, preventive treatment with melatonin could also reduce the risk of breast cancer induced in women by unidentified environmental factors.

## Introduction

It has been well documented that the pineal hormone, melatonin, besides its well established circadian rhythm, plays a major role in the control of reproduction in mammals [1,2]. The role of the pineal gland, the major source of melatonin, in the development of breast [3] and mammary [4,5] cancer has been clearly documented. Also, it has been shown that melatonin may exert *in vivo *[6,7] and *in vitro *[8-10] an oncostatic activity by at least a direct action on the mammary tissue [11] and on the activation of estrogen receptor (ER) for DNA binding [12], or by modulation of the expression of ER mRNA [13], or by an increase of the ER binding activity [14]. Recently, it was shown that melatonin acts as a calmodulin antagonist, inducing conformational changes in the ERα–calmodulin complex and thus impairing the binding of 17β-estradiol (E_2_) and the ERα–calmodulin complex to DNA and therefore preventing ERα-dependent transcription [15]. Also, 17β-estradiol treatment of pineal glands in an *in vitro *perifusion system leads to complete blunting of the isoproterenol-induced stimulation of melatonin secretion in 7,12-dimethylbenz [a]anthracene-treated (DMBA-treated) female Sprague–Dawley rats [16].

On the other hand, a single intragastric admininistration of DMBA has been shown to induce mammary tumors in young, cycling female Sprague–Dawley rats [17]. This carcinogen interacts with rapidly proliferating cells in the terminal end buds, forming DNA adducts, which in turn participate in transforming the normal terminal end bud cells to malignant pathways [18-20]. The susceptibility of Sprague–Dawley rats to DMBA is maximal at 55 to 60 days of age and is abolished by ovariectomy, suggesting the inducible action of the carcinogen depends on ovarian secretions [21]. Furthermore, during the latency period, estrous cycles are associated with blunted preovulatory surges of luteinizing hormone and follicle-stimulating hormone [22], an increased surge of 17β-estradiol [23], and disruption of the expression patterns of the genes for hypothalamic gonadotropin-releasing hormone and its pituitary receptor [24]. Moreover, ovariectomized rats pretreated with DMBA exhibit blunted release of luteinizing hormone in response to *in vivo *estradiol replacement, and reduced release of gonadotropin-releasing hormone as measured *in vitro *using synaptosomes from the mediobasal hypothalamus [25]

Considering the estrogenic properties of the DMBA molecule [26], it is possible that the carcinogen exerts its long-lasting effects at least on the plasma membrane of estrogen-sensitive neurons [27]. In addition, DMBA can interact with the ER and partially mimic both the positive and negative feedback actions of estradiol in ovariectomized rats [28].

The aim of this study was to compare the potency of melatonin to limit the frequency of cancer initiation with its potency to inhibit tumor progression once initiation by DMBA was achieved.

Our research team has previously shown that the administration of melatonin (10 mg/kg daily) for 6 months markedly reduces the percentage of tumor-bearing animals in DMBA-treated female rats, by 65% [16].

This study compared, over 1 year, the effect of preventive treatment with melatonin (10 mg/kg daily), administered for only 15 days before the administration of DMBA, to the effect of a curative treatment with the same dose of melatonin administered for 6 months after the administration of DMBA.

## Materials and methods

### Animals

Sixty 40-day-old female Sprague–Dawley rats (Charles River, L'Arbresle, France), 40 days of age, were used. They were housed (five animals per cage) under conditions of controlled temperature (20 to 22°C) and light (12 hours light /12 hours darkness; lights on from 0700 to 1900 hours). The animals had free access to commercial pelleted rat food (UAR, Villemoisson, Epinay-sur-Orge, France) and water. After 5 days of acclimatization, they were randomly assigned to three experimental groups of 20 animals each.

The first group of animals received a daily intragastric administration (in 1 ml of hydroxyethylcellulose at 1%) of melatonin (10 mg/kg daily, given no more than 3 hours before lights were turned off) for 15 days (from 45 to 59 days of age). At age 60 days, at noon on the day after cessation of the treatment, they were given a single intragastric administration of DMBA (Sigma, Saint Quentin Fallavier, France) (75 mg/kg) diluted in 1 ml of sesame oil, as described elsewhere [17].

The second group of animals received a single intragastric administration of DMBA (75 mg/kg) at noon when they were 55 days old. Then, beginning the next day, they received a daily intragastric administration of melatonin (10 mg/kg daily, given no more than 3 hours before the lights were turned off) for 6 months.

The third group of animals was given a single intragastric administration of DMBA (75 mg/kg, given at noon) at 55 days of age.

All the animals were followed up for 12 months after the administration of DMBA.

The occurrence of palpable mammary tumors was recorded every 2 weeks after the intragastric administration of DMBA.

### Tumor histology

The number and the size of mammary tumors were recorded and a histological analysis was performed. Tumors were removed when the largest diameter was at least 2 cm. They were dissected, trimmed free of surrounding connective tissue, and placed in 37% formaldehyde. Tumors were fixed in paraffin wax, sectioned, and stained with hematoxylin and eosin. Sections were examined by one of us (AM).

The lesions observed from the removed samples ranged widely from benign to malignant, with pathology closely resembling that seen in human breast tumors. The observed tumors were generally classified as adenocarcinomas (ADKs), with marked nuclear irregularities and numerous mitoses. Only clearly characterized ADKs were taken into account in the results presented in this study.

### Statistical analysis

The normality of data was analyzed with the Kolmogorov–Smirnov and Shapiro–Wilk tests.

As a preliminary step in the statistical process, a two-factor analysis of variance was performed: it was based on the comparison of means weighted by the time factor, because the classical underlying assumptions did not hold.

Because the samples were not drawn from gaussian populations, Student's *t *and the Welsh tests could not be used; therefore, the Mann–Whitney test was applied.

The percentages of animals diagnosed with mammary ADK in each data set were compared using the Fisher exact test (one-sided *P*-values).

## Results

### ANOVA two-way analysis

There was strong evidence (*P *< 0.01) that the ADK number increases within months after DMBA and that there was a very strong decrease of the number of ADK in treated rat groups (*P *= 0.01). In order to analyse these differences more closely, we looked to see whether the various time groups differed, whether any such difference was due to a decrease in the number of ADK-bearing animals, and whether the ADK number per ADK-bearing animals was reduced.

### Comparison of the mean numbers of mammary ADKs between the groups

**Figure 1:**
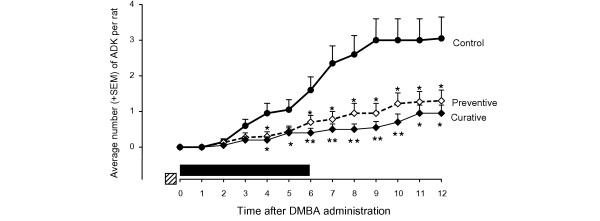
Average number (mean ± standard error of the mean) of mammary adenocarcinomas (ADKs) per rat after daily administration of melatonin (10 mg/kg) for either 15 days before (preventive treatment; open diamonds) or 6 months after (curative treatment; filled diamonds) the administration of dimethylbenz [a]anthracene (DMBA). In both cases, the rats were followed up for 12 months after the administration of DMBA. Hatched bar, duration of preventive treatment; filled bar, duration of curative treatment. *P < 0.05 vs controls; **P < 0.01 vs controls.

In control animals, there was an almost linear increase in the mean number of ADKs between the second and ninth months after DMBA administration (Fig. 1). The highest number (3.05 ± 0.59) was found 9 months after the administration of DMBA. In the melatonin-treated rats, there was a significant reduction of the number of ADKs in both the group given preventive treatment and that given curative treatment (Mann–Whitney test). Although the test used is nonparametric, the sample statistics mean and standard error of the mean are shown in Fig. 1 for a better description of the groups.

Twelve months after DMBA administration, values were 1.30 ± 0.30 in the the group given preventive treatment and 0.95 ± 0.23 in the group given curative treatment, vs 3.05 ± 0.59 in the control group (Fig. 1). Also, there was no statistical difference between the preventively and curatively treated groups (1.30 ± 0.30 vs 0.95 ± 0.23) (Mann–Whitney test). Nevertheless, the curative group differed more significantly from the control group than the preventive group (0.002 <*P *< 0.01), from 6 to 10 months.

### Percentage of rats with at least one palpable mammary ADK

**Figure 2:**
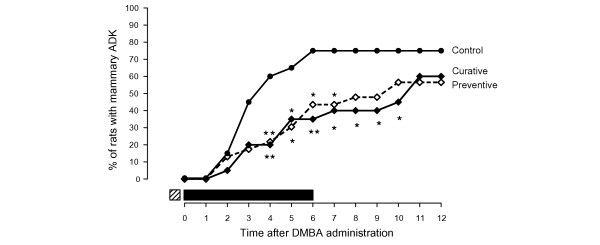
Incidence of mammary adenocarcinomas (ADKs) after daily administration of melatonin (10 mg/kg) for either 15 days before (preventive treatment; open diamonds) or 6 months after (curative treatment; filled diamonds) the administration of dimethylbenz [a]anthracene (DMBA). In both cases, the rats were followed up for 12 months after the administration of DMBA. Hatched bar, duration of preventive treatment; filled bar, duration of curative treatment. *P < 0.05 vs controls; **P < 0.01 vs controls.

In control animals, the first palpable mammary ADK (latency period) appeared 2 months after DMBA administration (Fig. 2). By 6 months after the administration of DMBA, 75% of control rats exhibited at least one palpable mammary ADK.

In animals that were pretreated daily with melatonin for either 15 days before the administration of DMBA or for 6 months after the administration of DMBA, there was a marked reduction in the percentage of animals with mammary ADKs as compared with the control group. Six months after DMBA administration, the ADK rate was 43% in the group given preventive treatment (*P *= 0.04 vs control group) and 35% in the group given curative treatment (*P *= 0.01 vs control group), vs 75% in the control group. The difference between the rates in the two experimental groups was not statistically significant. However, the inhibitory effect last significantly longer in the group given preventive treatment than in that given curative treatment (10 months vs 7 months, respectively).

### Comparison of the mean number of mammary ADK number between groups of ADK-bearing animals  (Fig. 3)

In control animals, between the fifth and the ninth month after DMBA administration there was an almost linear increase in the mean number of ADKs per ADK-bearing animal. At 9 months after administration of DMBA, the mean number (± standard error of the mean) was 4 ± 0.6 ADKs per ADK-bearing animal.

In the melatonin-treated female rats given preventive treatment, there was a reduction in the mean number of ADK per ADK-bearing animal but it did not reach a high degree of statistical significance.

The number of ADKs in the melatonin-treated female rats given curative treatment (1.38 ± 0.18 at 9 months after DMBA administration and 1.58 ± 0.26 at 12 months after DMBA administration) was significantly lower than in the control group (4 ± 0.59) (0.02 <*P *< 0.05 for month 7 to month 11 and 0.01 <*P *< 0.02 for month 12).

The differences between the preventive and the curative group (*P *> 0.1) and between the control and the preventive group (*P *≥ 0.05) were not statistically significant. However, the difference between the control and the preventive group was close or equal to 0.05 from month 7 to month 12.

## Discussion

The aim of this study was to compare the effect of daily intragastric administration of melatonin for 15 days before the administration of DMBA in the female rat with the effect of such treatment for 6 months after the administration of DMBA. Our results clearly show the preventive and curative effects of melatonin on the growth of DMBA-induced mammary ADK.

The curative effect of melatonin on the growth of DMBA-induced mammary ADK in the female rat has been previously well documented [4,5,16]. In the present study, the maximal inhibitory effect of melatonin on the percentage of rats with mammary ADK was by 62% at 4 months and 68% after 6 months of treatment (at the end of treatment). After the end of treatment, the intensity of the inhibitory effect started to decrease, but it was still significant up to 4 months after the end of treatment (10 months after DMBA administration).

The preventive (protective) effect of a brief period of melatonin treatment before DMBA administration has not been documented under the same experimental conditions. The maximal inhibitory effect of melatonin on the percentage of female rats with mammary ADK was a reduction to 62% relative to controls, at 4 months after DMBA administration. The Inhibition was significant up to 7 months after DMBA administration, but not later. Very similar results were obtained when the mean number of ADKs per rat or the mean number of ADKs per ADK-bearing rat were considered. Regarding the mean number of ADKs per ADK-bearing rat, there was a loss of significance, but there was no statistical difference between the effect of the preventive treatment and the curative treatment.

Interestingly enough, there was no statistical difference between the oncostatic activity afforded by melatonin according to whether it was administered by the preventive or the curative protocol: the incidence and number of ADKs were very similar, up to 7 months after DMBA administration. A major finding of this study is that 7 months after the induction of the carcinogenic process, both the percentage of animals with ADK and the mean number of ADKs per animal were still reduced in animals treated with melatonin for 15 days before DMBA administration.

In this study, the preventive treatment consisted of only 15 days of treatment, for technical reasons: the treatment could not be started before the rats were 40 days old, to avoid interference with the onset of estral cycling. A longer period of pretreatment with melatonin before the administration of DMBA might have led to a more pronounced inhibitory effect on the growth of DMBA-induced mammary ADK.

Many hypotheses have been proposed to explain the inhibitory effects of melatonin. They include modulation of the reproductive neuroendocrine axis (down-regulation of the gonadotropic axis and decrease of estrogen and prolactin levels); immunoenhancing activity; antioxidative properties; and direct antitumorigenic activity [8,9,29-32]. The direct oncostatic effect of metatonin has been mostly studied on *in vitro *models of estrogen-responsive breast cancer. In MCF-7 cells, melatonin interferes with the estrogen response pathway through the suppression of ERα expression and transactivation to affect the level of growth regulatory genes mediating the mitogenic action of estradiol [33,34]. Also, melatonin inhibits EGF and MAPK-induced cell proliferation via the suppression of linoleic acid uptake and metabolism [35] and it promotes cell differentiation (differentiation of terminal end buds) by reducing the invasiveness of MCF-7 cells and increasing gap functional contacts [35]

It is thought that DMBA acts, at the mammary gland level, throughout the formation of a cascade of metabolites (generated at least in the liver and the mammary gland) which ends with its ultimate carcinogenic form, the 3,4-dihydro-diol-1,2-epoxide, before adduct formation with DNA occurs [36,37]. Melatonin may inhibit adduct formation with DNA because of its free radical scavenging/antioxidant action [38]. In that view, it may be envisaged that the strong artificial amplification of the intensity of the circadian rhythm of melatonin, provoked by an exogenous supply, before the administration of DMBA could play a major preventive role against the induction of the carcinogenic process [39].

In the group of rats given long-term melatonin treatment beginning a day after DMBA administration, DMBA metabolism and adduct formation certainly still occur for at least 5 days (unpublished results). Therefore, the results obtained in the curative group represent the sum of an inhibition of both the initiation and the promotion process. Obviously, if the melatonin treatment had started a week or two after DMBA administration, we might have seen different results. However, in another carcinogen model of mammary tumorigenesis – the *N*-nitrosomethylurea model of hormone-responsive rat mammary carcinogenesis – melatonin was without effect on carcinogenesis when its administration was restricted to the initiation phase but was quite effective during the promotion phase [40].

Also, changes in circadian rhythms have already been documented to be associated with carcinogenesis [41,42], and it has been shown that the administration of melatonin could restore the deficiency of the circadian clock provoked by the low secretion of melatonin induced by DMBA [16]. Therefore, it can be envisaged that amplifying the intensity of the circadian rhythm of melatonin might help to prevent the induction of the carcinogenic process by DMBA.

## Conclusion

In view of the present results obtained in the female Sprague–Dawley rat and of the well-documented oncostatic properties of melatonin, it can be envisaged that the long-term inhibition of mammary ADK promotion by a brief preventive treatment with melatonin could also reduce the risk of breast cancer induced in women by unidentified environmental factors.

## Abbreviations

ADK = adenocarcinoma; DMBA = dimethylbenz [a]anthracene; ER = estrogen receptor.

## Competing interests

The author(s) declare that they have no competing interests.

## Authors' contributions

VL carried out the experimental studies and performed a detailed analysis of the data. MBYDJC equally carried out the experimental studies. MHP made a contribution to the acquisition of data. AM performed the histological analysis. RS participated in the design of the study and performed the statistical analysis. BK conceived the study and participated in its design and coordination and helped to draft the manuscript. All authors read and approved the final manuscript.

## Figures and Tables

**Figure 3:**
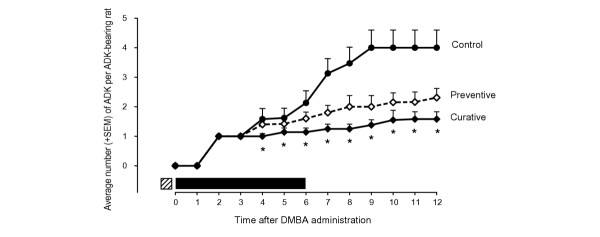
Average number (mean ± standard error of the mean) of mammary adenocarcinomas (ADKs) per ADK-bearing rat after daily administration of melatonin (10 mg/kg) for either 15 days before (preventive treatment; open diamonds) or 6 months after (curative treatment; filled diamonds) the administration of dimethylbenz [a]anthracene (DMBA). In both cases, the rats were followed up for 12 months after the administration of DMBA. Hatched bar, duration of preventive treatment; filled bar, duration of curative treatment. *P < 0.05 vs controls; **P < 0.01 vs controls.
